# Nicotinergic Modulation of Attention-Related Neural Activity Differentiates Polymorphisms of DRD2 and CHRNA4 Receptor Genes

**DOI:** 10.1371/journal.pone.0126460

**Published:** 2015-06-16

**Authors:** Thomas P. K. Breckel, Carsten Giessing, Anja Gieseler, Sarah Querbach, Martin Reuter, Christiane M. Thiel

**Affiliations:** 1 Biological Psychology, Department of Psychology, European Medical School, Carl-von-Ossietzky Universität Oldenburg, Oldenburg, Germany; 2 Research Center Neurosensory Science, Carl-von-Ossietzky Universität Oldenburg, Oldenburg, Germany; 3 Personality & Biological Psychology, Department of Psychology, University of Bonn, Bonn, Germany; 4 Cluster of Excellence “Hearing4all”, Carl von Ossietzky Universität Oldenburg, Oldenburg, Germany; 5 Center for Economics & Neuroscience (CENs), Laboratory of Neurogenetics, University of Bonn, Bonn, Germany; Leibniz Institute for Neurobiology, GERMANY

## Abstract

Cognitive and neuronal effects of nicotine show high interindividual variability. Recent findings indicate that genetic variations that affect the cholinergic and dopaminergic neurotransmitter system impact performance in cognitive tasks and effects of nicotine. The current pharmacogenetic functional magnetic resonance imaging (fMRI) study aimed to investigate epistasis effects of CHRNA4/DRD2 variations on behavioural and neural correlates of visuospatial attention after nicotine challenge using a data driven partial least squares discriminant analysis (PLS-DA) approach. Fifty young healthy non-smokers were genotyped for CHRNA4 (rs1044396) and DRD2 (rs6277). They received either 7 mg transdermal nicotine or a matched placebo in a double blind within subject design prior to performing a cued target detection task with valid and invalid trials. On behavioural level, the strongest benefits of nicotine in invalid trials were observed in participants carrying both, the DRD2 T- and CHRNA4 C+ variant. Neurally, we were able to demonstrate that different DRD2/CHRNA4 groups can be decoded from the pattern of brain activity in invalid trials under nicotine. Neural substrates of interindividual variability were found in a network of attention-related brain regions comprising the pulvinar, the striatum, the middle and superior frontal gyri, the insula, the left precuneus, and the right middle temporal gyrus. Our findings suggest that polymorphisms in the CHRNA4 and DRD2 genes are a relevant source of individual variability in pharmacological studies with nicotine.

## Introduction

Nicotine acts as stimulant of the cholinergic system and plays an important role in cognitive functions, especially attentional processing [1, 2]. Genetic factors contribute to both, individual differences in the cognitive effects of nicotine and smoking behaviour [3, 4]. The majority of research has focussed on genetic variations related to nicotinic cholinergic receptors and reward processing since nicotine binds to nicotinic receptors in the ventral tegmental area (VTA) and striatum leading to increased dopamine release [5].

Genetic variants in the genes coding for the α4 subunit of the cholinergic nicotinic receptor (CHRNA4) or the dopaminergic D2 receptor (DRD2) are associated with smoking behaviour on the one hand and with visuospatial attention and memory processes on the other hand which may be relevant for personalized treatment of nicotine addiction and cognitive dysfunction [4, 6]. A single nucleotide polymorphism in the CHRNA4 gene (rs#1044396), which codes for a protein subdomain in the α4β2 ACh receptor, has been related to performance in a variety of attentional tasks including multiple object tracking, visual search and cued target detection [7–10]. In contrast, allelic variants in the DRD2 gene (SNP rs#6277), which mediate D2 receptor density in the brain [11, 12], have been associated with working memory [13–15], impulsivity [16]; everyday cognitive failure [17] and performance in the Wisconsin Card Sorting Test [18]. Recently, two studies by Markett and colleagues provide however evidence for epistasis effects (gene by gene interaction effects) of DRD2 and CHRNA4 polymorphisms on working memory performance and striatal gray matter volume [14, 19].

While the above studies demonstrate that attention and working memory are modulated by variations in cholinergic and dopaminergic receptor genes, only few studies investigated, whether the often reported variability of cognitive effects of nicotine (see [2] for review) is influenced by variations in both, CHRNA4 and DRD2 genes. One study investigated how variations in the APOE4 gene are related to reorienting-related brain activity under nicotine and found modulations of neural activity in anterior cingulate cortex and parahippocampus [20]. Genotype dependent effects of nicotine were further shown for the DRD2 gene (rs#6277) with respect to working memory performance and neural activity in anterior insula and visual areas [21]. In addition, a polymorphism in the vicinity of the DRD2 gene (DRD2/ANKK1 TaqIa) that is in linkage disequilibrium with the DRD2 rs#6277 polymorphism was found to modulate the behavioural effects of nicotine in a spatial attention task with emotional distractors [22].

The genetic neuroimaging studies by Markett et al. [19] and Jacobsen et al. [21] suggest that DRD2 polymorphisms impact brain structure and working memory related neural activity in interaction with nicotinic polymorphisms or stimulation of the nicotinic neurotransmitter system respectively pointing to a dynamic interaction between cholinergic and dopaminergic neurotransmitter systems. We here aimed to investigate how epistasis effects of DRD2 and CHRNA4 variation modulate the stimulant effects of nicotine on reorienting visuospatial attention and related brain networks. A visuospatial cueing paradigm (Posner-paradigm [23]) was used in a double-blind placebo controlled fMRI study with nicotine to test if the often reported behavioural and neuronal effects of nicotine (e.g. [24]) depend on CHRNA4 and DRD2 genes. We employed a multivariate data analysis approach which identifies distributed patterns of brain activity because changes in neurotransmitter systems induced by genetic variations or drugs affect brain networks rather than isolated brain regions. The aim was to identify, whether a specific pattern of nicotine-induced reorienting-related brain activity can differentiate between genotypes.

## Material and Methods

### Subjects

Fifty healthy, right-handed, and non-smoking subjects (33 female, 17 male; mean age = 23.84 years, range = 19 to 37 years) were recruited from the student population of the University of Oldenburg. Three subjects were excluded from fMRI data analysis due to head motion. The study was approved by the ethics committee of the German Psychological Association and subjects signed written informed consent.

### Genotyping

The polymorphisms in the CHRNA4 and DRD2 genes were genotyped based on the DNA of buccal cells by means of real time PCR (Light Cycler System, Roche Diagnostics, Mannheim, Germany) using fluorescent probes besides conventional primers (TIB-MOLBIOL, Berlin, Germany). Analyses focussed on the single nucleotide polymorphisms (SNP) rs#6277 on the DRD2 gene and the SNP rs#1044396 on the CHRNA4 gene. An automated purification of genomic DNA was conducted by means of the MagNA Pure LC system using a commercial extraction kit (MagNA Pure LC DNA isolation kit; Roche Diagnostics, Mannheim, Germany).

### Experimental procedure and paradigm

Subjects participated in two separate, double-blind placebo controlled fMRI sessions. Each session was separated by approximately one week. Nicotine was administered by a 7 mg nicotine patch (Niquitin 7mg patch, GlaxoSmithKline Consumer Healthcare GmbH), a customary band-aid (transparent and tailored to the size of the nicotine patches) was used as placebo. Patches were placed on the back of the subjects above waistline and covered by a larger, opaque patch. The patches were applied 50 minutes before the experimental sessions and removed before subjects entered the MR-scanner.

**Fig 1:**
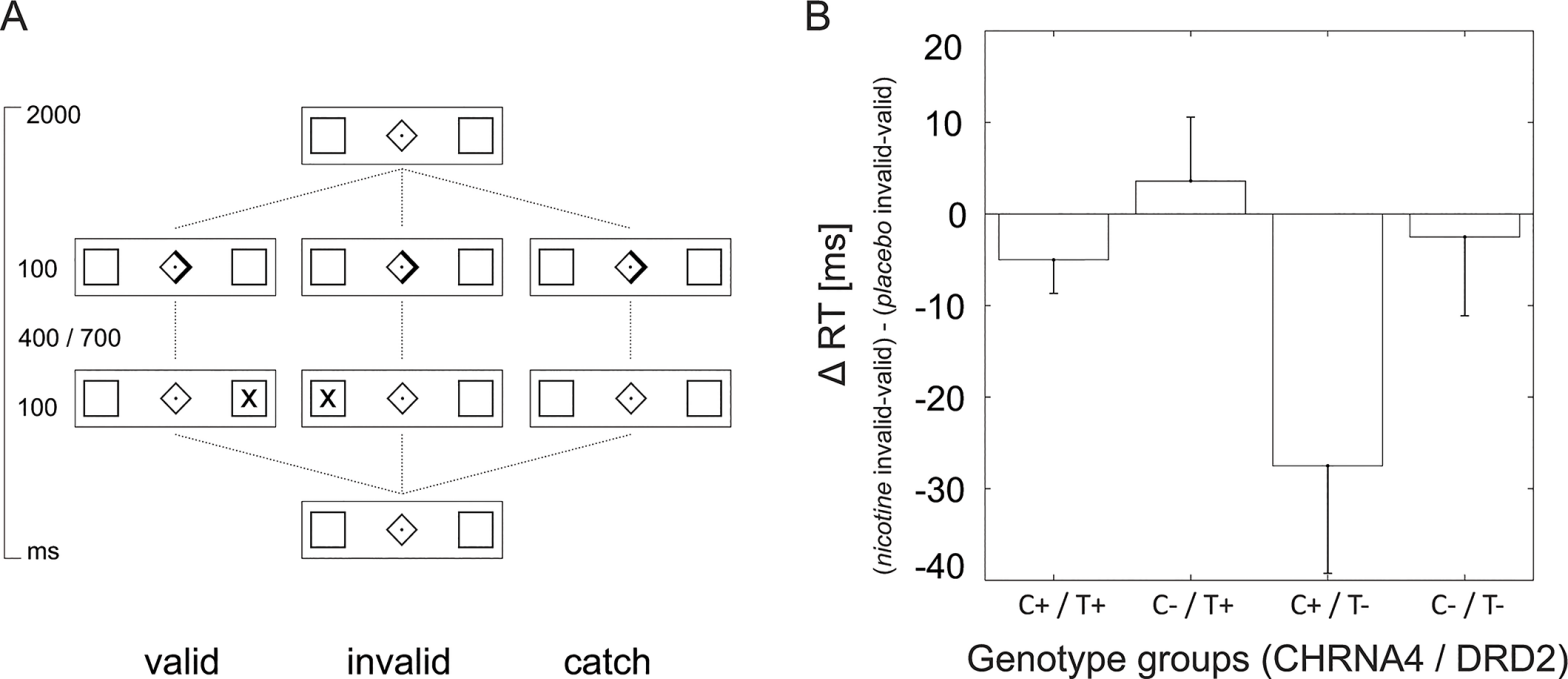
Visual cueing paradigm (A) and behavioural results (B). **A.** Scheme of the three task conditions; valid (120), invalid (30), catch (20), and zero (50, no cue and no target, not depicted) trials were presented in randomized order with a SOA of 2000 ms. **B.** Difference of the validity effect (slowing of RTs due to invalidly cued trials as compared to valid trials) between nicotine and placebo. The CHRNA4 C+ and DRD2 T- genotype group shows a significant benefit from nicotine. The significant three-way interaction of *genotype group* x *treatment* x *condition* as displayed in the current figure was identified by post-hoc ANOVAs to be driven by the *genotype group* x *treatment* interaction during invalid trials.

In the MR-scanner subjects performed two visuospatial attention tasks separated by a break of 7 minutes. The relevant task for the current study was a visual cueing paradigm (Posner-Paradigm) and was the second of the two tasks (after 35 minutes from the start of the scanning session). The visual cueing paradigm consisted of 220 trials with valid (120), invalid (30), catch (20, cue only) and zero (50, no-cue no-target) trials presented in random order with a stimulus onset asynchrony (SOA) of two seconds (see Fig 1A). The cue stimulus was presented for 100 ms and the cue-target interval was 400 or 700 ms, balanced over all trials. The target stimulus (letter X) was presented for 100 ms in one of the two peripheral boxes to the left or to the right (9.6° eccentric in each visual field). Subjects were instructed to fixate the centre of the screen and to respond as fast as possible to target appearance with a left or right button press according to the target’s location (right hand, index finger and middle finger). The stimulus presentation was programmed with the Cogent Graphics v. 1.32 Toolbox (www.vislab.ucl.ac.uk) for Matlab (The MathWorks, Inc.).

Blood pressure and heart rate were measured before patch application (t1), shortly after the patch was removed (t2), and after task completion outside the MR cabin (t3). At the same three time points, the subjects were further asked to rate subjective physical symptoms (ten items, from ‘not existent’ to ‘very strong’) and mood by means of the mood rating scale by Bond and Lader [25].

### Analysis of behavioural data

Reaction times (RT) of valid and invalid trails were analysed with an ANOVA for repeated measurements modelling the main effects of condition, treatment, genotype group and their interactions. The error rate was low (placebo 2.6 ± 0.7% and nicotine 2.1 ± 0.3% errors with SEM) and thus not subject of further analysis.

### FMRI data acquisition

Functional and structural images were acquired on a 3.0 Tesla MRI scanner (Siemens MAGNETOM Verio, Siemens AG, Erlangen, Germany). Functional images were obtained using a multislice T2*-weighted gradient echo planar imaging method (EPI). Each volume consisted of 27 axial slices (voxel size of 3 x 3 mm, 3 mm in slice thickness, slice gap of 10%, FoV = 200 x 200 mm^2^, TR = 1500ms, TE = 30 ms and 80° flip angle, ascending axial). During the spatial cuing paradigm 294 whole brain scans (7 min 56 s) were acquired. Structural T1-weighted images were obtained after the experiment, using magnetization frequency pulse and rapid gradient-echo (MP RAGE) sampling (1 mm isotropic, FoV = 256 x 256 mm^2^, TR = 1900 ms, TE = 2.52 ms and 9° flip angle, sagittal).

### FMRI data processing and first level analysis

FMRI data were preprocessed using SPM8 (FIL, Welcome Trust Centre for Neuroimaging, UCL, London, UK). Functional images were spatially realigned to compensate for subjects’ head movements, coregistered with the structural image, normalized to MNI space based on the segmented structural image and the grey and white matter MNI-templates of SPM8, and spatially smoothed with a three dimensional isotropic Gaussian filter of 8 mm full-width-at-half-maximum. Three subjects had to be removed from further data analysis because of extensive head motion (more than five scan-to-scan displacements above 0.5 mm or a total drift of more than 3 mm) in one or both of the scanning sessions (47 subjects remained for fMRI data analysis and the PLS-DA).

First level statistical analysis based on an event-related design with regressors coding for each of the trial types (valid, invalid and catch trials) and one regressor for missed trials or trials with false responses. The six estimated motion parameters from the spatial realignment were added to the model in order to code for signal changes related to head motion. Both treatment conditions, nicotine and placebo, were considered in a joint first level model with two sessions. After model estimation, the individual differences of BOLD responses of the contrast ‘*invalid nicotine* minus *invalid placebo’* were used as data basis for the PLS-DA approach. The data were also used to illustrate the directionality of the reported effects (Table 1). For the post-hoc testing the mean BOLD responses of the brain regions identified by the PLS-DA approach (see below) were tested with two-sided T-tests for each genotype group separately.

### Partial least square discriminant analysis (PLS-DA)

Goal of the PLS-DA approach was to predict the four genotype groups with the voxel-wise BOLD signal changes due to the pharmacological treatment. Partial least square (PLS) regression is a multivariate statistical approach for data prediction and is used when the predictor variables are high in number and highly collinear [26–28]. It is a dimension reduction technique that extracts latent variables (LVs) which account for maximal covariance between response and predictor space; for comparison, principal component analysis optimizes for maximal variance in the predictor space only. PLS-DA is a variant of PLS regression which can be used to predict categorical instead of continuous variables (e.g. see [29]). In PLS-DA, the response space is represented by a ‘dummy’ matrix coding column wise for each class (in our case 47 rows for the subjects, 4 columns for the genotype groups). The predictor variables consisted of the voxel-wise extracted beta values from the contrast ‘*invalid nicotine* minus *invalid placebo* trials’ of all subjects (47 rows for the subjects, columns for the voxels). In a first ‘whole brain’ model we calculated the ‘variable importance in prediction (VIP)’ values [30, 31] of each voxel, based on all gray matter voxels in the measured field of view (FoV) of all subjects intersected with the AAL-template from the wfu-pickatlas toolbox for SPM8 [32] (105,476 voxels in total). The VIP values were calculated for 500 bootstrap samples in order to minimize potential biases by single subjects or a single genotype group. A second PLS-DA model was used for classification and based on the 5% most important predictor variables (5,274 voxels) which were equivalent to voxels with a mean VIP value ≥ 1.28 (see also S1 Fig).

To test the significance of each LV extracted by the model we used a leave-out-one-sample (LOO) cross validation procedure combined with the van der Voet model comparison test [33]. Thereby the root mean predicted residual sum of squares (MPRESS; [34, 35]) was calculated for different models in which successively LVs were added. Only LVs that significantly improved the model’s prediction (p≤0.05, based on the randomization test of van der Voet, 1994) were used for classification of the genotype groups. For classification each subject was grouped according to their highest predicted probability of group membership. For single subjects, the classification uncertainty was calculated with the Euclidean distance between the class representation on the response side (dummy coded with ‘1’ and ‘0’) and the predicted values during cross validation. For calculation of the PLS models the SIMPLS algorithms of Dejong [36] and Rosipal and Kramer [37] were used as implemented in the Statistics Toolbox of Matlab v7.8 (R2009a, The Mathworks Inc.). To investigate whether age and gender had an impact on classification accuracy we tested the classification uncertainty of correctly classified subjects for correlation with age (Pearson’s r) and differences between females and males (Mann-Whitney-U-test).

## Results

### Genotypes

The observed allele frequencies in the two genes matched the distribution as expected from the Hardy-Weinberg theorem (allele configuration with number of subjects: CHRNA4 C/C (9), C/T (23), T/T (16), Chi² p = 0.88; DRD2 T/T (12), T/C (22), C/C (14), Chi² p = 0.57). Different allele carriers were then assigned to one of four genotype groups following the rationale of Markett and colleagues (2010, 2013) to investigate epistatic effects. For grouping, carriers of the CHRNA4 C allele (CHRNA4 C+ = C/C and C/T versus CHRNA4 C- = T/T) and carriers of the DRD2 T allele (DRD2 T+ = T/T and T/C versus DRD T- = C/C) were pooled (further details for this grouping see discussion part). The genotype groups in the current study reflect the resulting four possible genotype configurations of both genes: [CHRNA4 C+ and DRD2 T+, 27 subjects in total, 24 subjects for fMRI/PLS-DA analysis], [CHRNA4 C- and DRD2 T+, 9 subjects], [CHRNA4 C+ and DRD2 T-, 7 subjects], and [CHRNA4 C- and DRD2 T-, 7 subjects]. Age did not differ between the four groups (F(3,43) = 0.94, p = 0.43). There were also no differences in gender distribution across groups (χ = 2.01, df = 3 p = 0.57).

### Behavioural Data

Subjects responded slower to invalid as compared to valid trials (*condition*: 438 (±6) vs. 356 (±5) in ms ±SEM, F(1,46) = 356.75, p<0.001). Nicotine treatment lead to faster responses (*treatment*: 393 (±8) vs. 400 (±9) in ms ±SEM, F(1,46) = 5.93, p<0.05) and reduced the validity effect (*condition x treatment* F(1,46) = 4.29, p<0.05; validity effect nicotine 79 (±5) vs placebo 85 (±5) in ms ±SEM,). Genetic variations showed no main effect on RTs (*genotype group* F(3,46) = 0.30, p = 0.83) nor an interaction with treatment (*treatment x genotype group* F(3,46) = 2.35, p<0.1) but showed a significant interaction with condition (*condition x genotype group*, F(3,46) = 3.38, p<0.05) and importantly a genotype x condition x treatment interaction (F(3,46) = 2.92, p<0.05). This interaction was driven by CHRNA4 C+ / DRD2 T- carriers who showed a significant reduction of the validity effect under nicotine (Fig 1B and S1 Table). Note, that we did not find any main effect of genotype nor interaction with condition and treatment when considering each SNP alone (SNPs of CHRNA4 or DRD2 only with 3-level factors CHRNA4 genotype x condition x treatment F(2,47) = 1.58 p = 0.22, DRD2 genotype x condition x treatment F(2,47) = 1.57 p = 0.22). Two separate post-hoc ANOVAs on the valid and invalid trials showed a *treatment* effect (F(1,46) = 7.90, p<0.01) and a significant *genotype group x treatment* interaction (F(3,46) = 3.672, p<0.05) for invalid trials. In contrast, the ANOVA for valid trials did not show any significant effects (*treatment* F(1,46) = 1.89, p = 0.18, *genotype group x treatment* F(3,46) = 0.56, p = 0.64). This finding indicates that the *condition x genotype x treatment* interaction was driven by differential effects of nicotine in *invalid* trials only. Because of the finding that genotype dependent effects of nicotine treatment on behaviour were only present in the invalid task condition, we subsequently focussed the PLS-DA approach on BOLD response differences between nicotine and placebo treatment in the invalid condition.

To gauge physiological drug effects, measures of heart rate and blood pressure (mean arterial pressure) were tested over time under nicotine and placebo. We found significant main effects and interactions for heart rate (time point F(2,88) = 22.11, p<0.01; treatment F(1,44) = 6.56, p<0.05; time point x treatment interaction F(2,88) = 4.69, p<0.01). The time point x treatment interaction reflects a significantly higher heart rate after nicotine (t2, p<0.01 and t3, p<0.05) as compared to placebo. No effects of time point or treatment were found for the mean arterial pressure.

### Neural data: Partial least squares discriminant analysis (PLS-DA)

**Fig 2:**
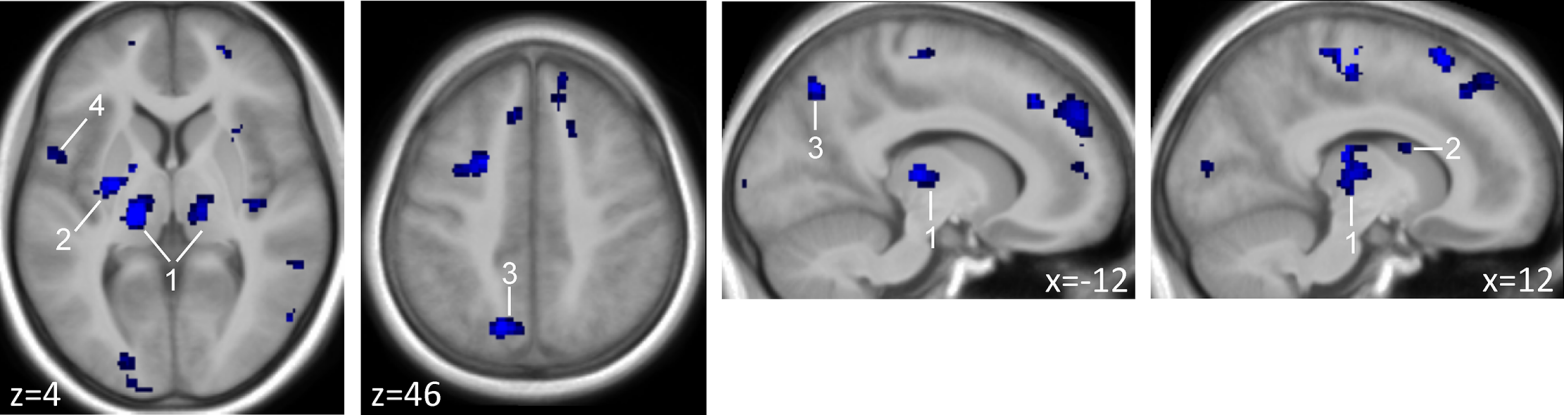
Identified brain regions that were most relevant for the differentiation of CHRNA4 and DRD2 polymorphisms. The PLS-DA based on the contrasted BOLD estimates between the nicotine and placebo treatment sessions during invalid trials. The nicotine-induced main variance in these regions was able to successfully predict the four genetic groups (see Fig 4). Regions are displayed at VIP threshold ≥ 1.28 (see methods section), cluster extent threshold k≥40. (1) Pulvinar nuclei of the thalamus, (2) putamen/striatum, (3) precuneus, (4) insula; see Table 1 for a complete list.

We used the PLS-DA approach to identify brain regions that showed genotype-dependent differences in BOLD responses in invalid trials under nicotine as compared to placebo. Since the behavioural data suggest epistatic rather than single gene effects we focussed our analysis of genotype-dependent neural effects of nicotine on the combined genotype groups. In a first step (VIP filtering) we identified a network of brain regions such as the pulvinar nuclei of the thalamus, the striatum, the middle and superior frontal gyri, the insula, the left precuneus, and the right middle temporal gyrus (see Fig 2 and Table 1). Note, that these brain regions are similar to those obtained by a conventional SPM ANOVA model with genotype group as a 4-level factor (see S2 Fig).

**Table 1:** Identified brain regions contributing to genotype classification.

Region	MNI (mm)^1^			Size (voxel)^2^	BOLD modulation by nicotine in CHRNA4/DRD2 groups^3^			
					C+ / T+	C- / T+	C+ / T-	C- / T-
Superior Frontal Gyrus, left	-14	50	30	411	0	0	0	0
Superior Frontal Gyrus, right	16	22	56	147	0	0	0	0
Medial Frontal Gyrus, left	-8	-28	60	55	0	0	-	0
Medial Frontal Gyrus, right	12	38	46	77	0	[-]	0	0
Middle Frontal Gyrus, left	-36	50	14	97	[-]	+	0	0
	-32	48	-4	67	-	+	0	0
	-28	4	46	60	0	0	0	0
Middle Frontal Gyrus, right	36	42	14	222	0	+	+	0
	32	14	58	53	[-]	[+]	[+]	0
	22	56	24	45	+	0	0	0
Inferior Frontal Gyrus, left	-28	-30	-6	56	0	0	-	0
Precentral Gyrus, left	-42	-12	58	49	-	0	0	0
Supplemental Motor Area, right	8	-22	58	148	0	0	-	-
Inferior Parietal Lobule, right	50	-38	34	64	-	+	0	0
Middle Temporal Gyrus, right	46	-74	20	126	0	-	0	0
	58	-22	-8	114	-	0	+	0
	60	-46	0	75	[-]	[+]	+	0
Cuneus, left	-20	-92	4	66	0	0	-	0
Cuneus, right	14	-86	10	46	0	+	-	0
Precuneus, left	-12	-70	46	126	-	+	0	0
Superior Occipital Cortex	24	-72	20	101	0	0	0	0
Amygdala, left	-22	-4	-16	65	-	+	0	0
Rolandic Operculum, left	-42	6	12	367	-	+	0	0
Rolandic Operculum, right	46	-14	20	146	-	+	0	0
Insula, left	-38	-4	-10	61	[-]	+	0	0
Insula, right	44	6	-10	348	0	+	0	0
Insula, right	38	22	14	48	0	[-]	0	0
Striatum (Caudate Nucleus), right	16	6	20	43	[-]	0	0	0
Striatum (Putamen), left	-26	-8	2	210	-	+	0	0
Striatum (Putamen), right	30	2	10	183	-	+	0	0
Thalamus (Pulvinar), left	-14	-20	4	236	-	+	0	0
Thalamus (Pulvinar), right	12	-20	8	209	-	+	0	0
Hippocampus, right	36	-24	-10	94	0	0	-	0

^1^ Center of gravity coordinate of the clusters.

^2^ Only clusters that exceeded the voxel extent threshold of k≥40 are reported.

^3^ Clusters showing increased (+) or decreased (-) BOLD levels under nicotine (p≤0.05) for each genotype group (CHRNA4 / DRD2) during invalid trials. Post-hoc tests of mean cluster BETA values; tendencies (p≤0.1) are indicated by rectangle brackets.

**Fig 3:**
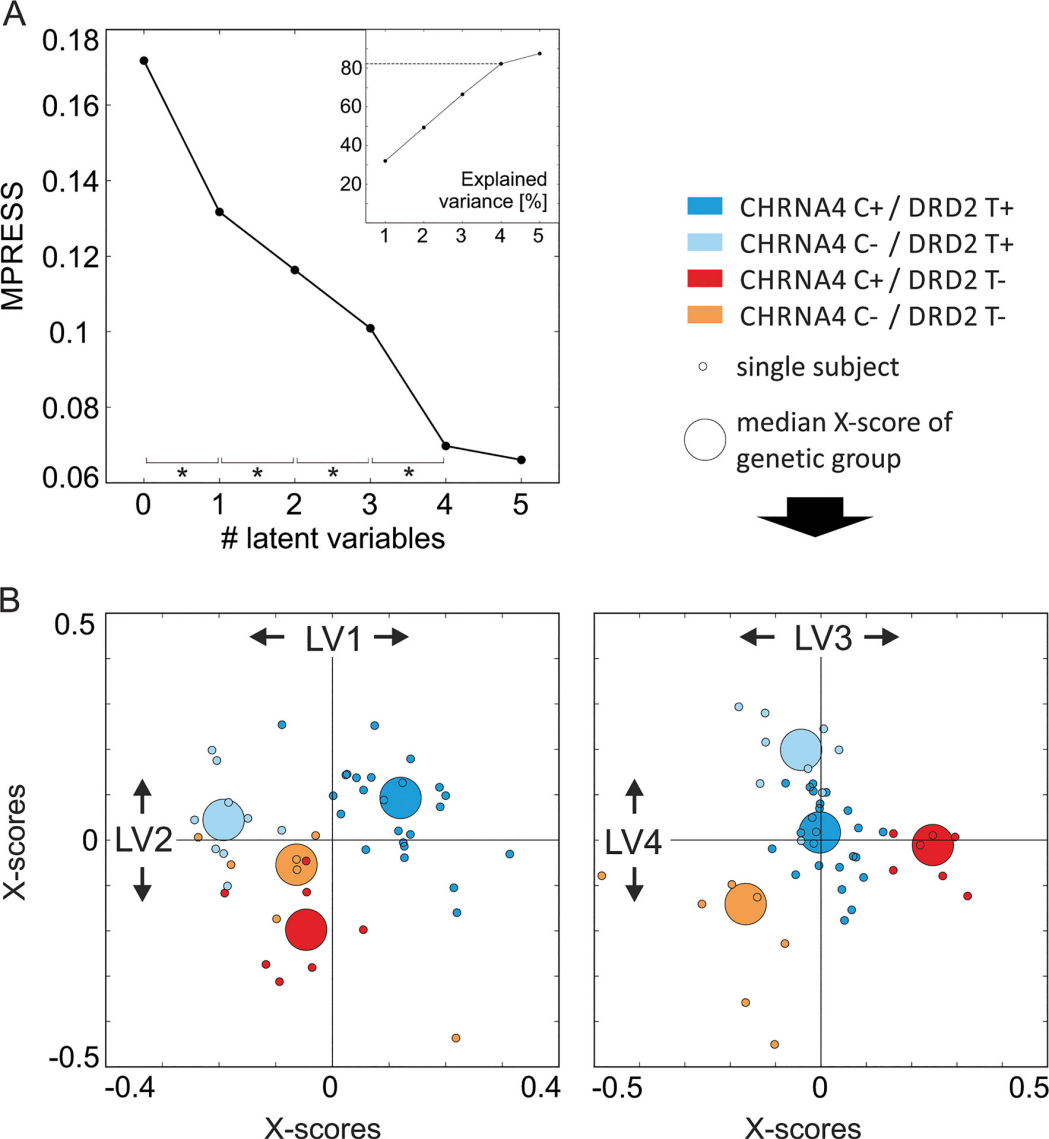
A. Residual errors and explained variance by the latent variables (LVs) of the PLS-DA model. The PLS-DA model derived four significant LVs from the selected voxels (VIP≥1.28 as depicted in Fig 2, p-values of LVs≤0.05). Voxel values based on the treatment evoked variance of the BOLD responses during the invalid task condition. The four LVs explained in total 82.30% of the model’s variance (inlay upper right corner) and were used for classification of the genotype groups. **B. X-scores of the latent variables (LVs).** Descriptive plot to visualize the discrimination power of the four significant LVs of the current PLS-DA model. The X-scores are the sum of the weighted predictor variables (voxel values) for each LV and depicted here for each subject (small dots). For illustration, the colour code is set to the true genotype group of each subject, large dots represent the median X-score of each group. The plot shows how the PLS-DA model projects the original X data space in order to optimize the covariance between the genotype groups and X-scores. It is visible that, for example, the first LV (x-axis of the left plot) differentiates well between the prevalent CHRNA4 C+ / DRD2 T+ carriers (coloured in darker blue, positive X-scores for LV1) and the other three genotype groups (negative X-scores for LV1). The third LV (x-axis of the right plot) differentiates well between e.g. the third genotype group, which showed the behavioural benefits of nicotine (red, positive X-scores for LV3) and the fourth genotype group, (orange, negative X-scores for LV3), but not between the first two genotype groups (blue colours, X-scores around zero). Note that the information of all four LVs is used in the PLS-DA classification to predict the genotype group of the subjects.

In a second step, we extracted four significant LVs (p≤0.05) based on the prediction from the above brain regions that explained 82.30% of the model’s variance (see Fig 3A). The discrimination power of each LV can be illustrated with the calculation of the estimated X-scores for each LV (Fig 3B). Note that the LVs are not independent from each other and reflect a three-way interaction between BOLD responses in invalid trials under placebo and nicotine, genetic grouping and attributed weights from the model’s prediction. Table 1 depicts genotype dependent increases and decreases under nicotine in the identified brain regions. These findings show that subjects with the prevalent s CHRNA4 C+ / DRD2 T+ genotype mainly show reductions of BOLD activity in parietal, frontal, and temporal brain regions in invalid trials under nicotine, i.e. the effect reported in many prior studies [24, 38–40].The findings further suggest that overall BOLD modulation under nicotine is generally stronger (independent of its direction) in subjects with low dopaminergic activity (i.e. DRD T+). The CHRNA4 C+ / DRD2 T- group, which showed a behavioural benefit of nicotine was characterized by increases rather than decreases in BOLD activity in right middle frontal and right middle temporal gyrus, previously found to be downregulated under nicotine (e.g. [39]).

**Fig 4:**
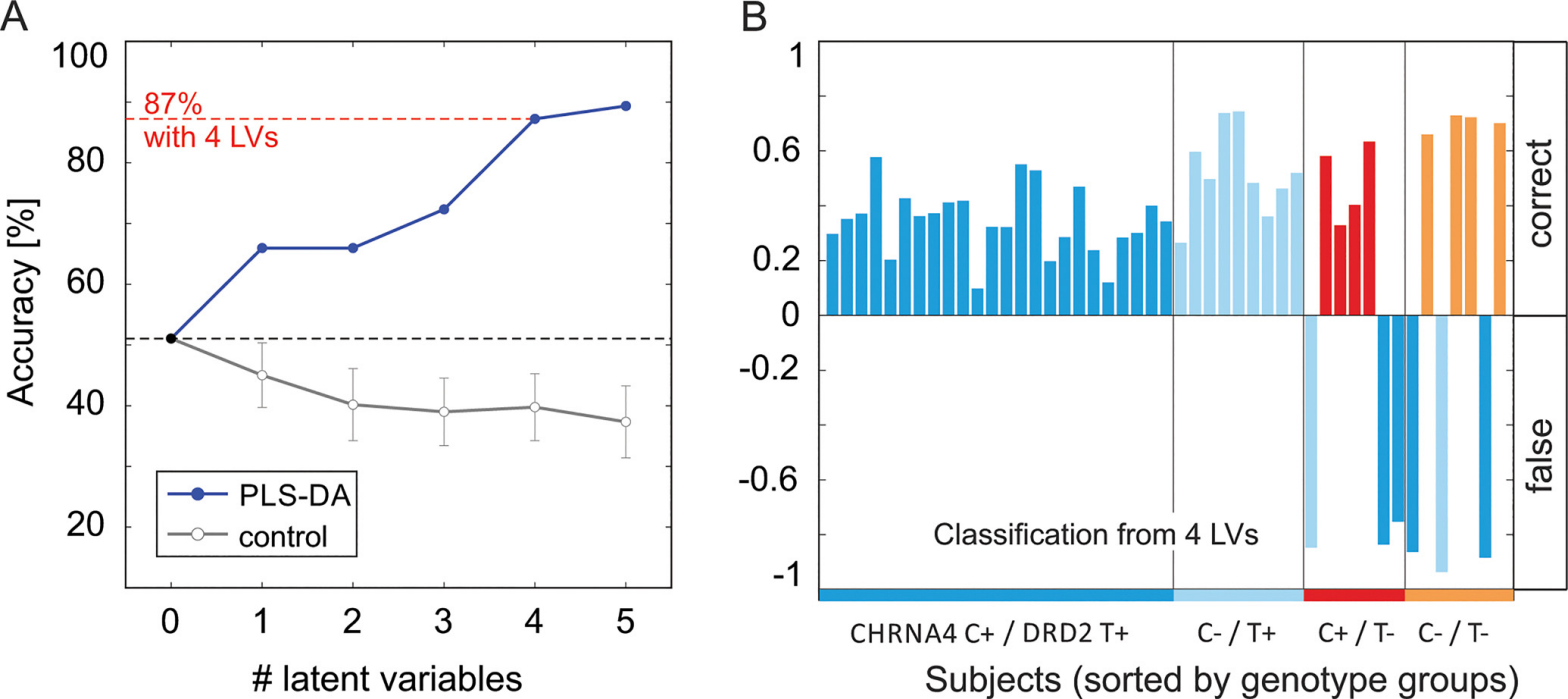
A. Classification accuracy. Prediction accuracy successively increases with adding LVs (blue). With four LVs, 41 out of 47 subjects (87.23%) were successfully assigned to their genetic group. When using no LV, all subjects were assigned to the genetic group with the highest frequency, which is the CHRNA4 C+ / DRD2 T+ group with n = 24 (51.06%). When randomizing the genotype groups among the subjects (control with SEM, repeated 50 times) the accuracy even drops below chance level which indicates that the PLS-DA models based on randomized groups only add noise to the prediction of the ‘null’ model. **B. Uncertainty over subjects.** Classification uncertainty over subjects based on the Euclidean distance between the predicted values and the dummy coded representation of the genotype group. Correct classifications are depicted as positive values, false classification as negative values. The grouping on the x-axis gives the true genotype groups of the subjects (together with the colour code). The bar colours give the assigned genotype group from the PLS-DA. Falsely classified subjects were present in the two smaller genotype groups (C+/T- and C-/T-).

The prediction accuracy of the PLS-DA model improved with successively adding further LVs (see Fig 4A). With the first four, significant LVs 41 out of 47 subjects (87.23%) could be correctly assigned to their genetic group based on nicotine-induced BOLD responses in invalid trials. Estimates about the classification uncertainty for each subject are given in Fig 4B. Classification uncertainty was lower for subjects in the largest group (CHRNA4 C+ / DRD2 T+ group, prediction error range 0.1 to 0.59) and larger for the smaller groups (range 0.21 to 0.9). Falsely classified subjects were found in the two smallest groups only (CHRNA4 C+ / DRD T- and CHRNA4 C- / DRD2 T- groups). The accuracy of classification was not different between males and females (uncertainty female = 0.42 (+/-0.03 SEM), uncertainty male = 0.45 (+/- 0.04 SEM), Mann-Whitney-U-test p = 0.57), nor did it correlate with age (R² = 0.04, p = 0.19; uncertainty of correct classified subjects only).

To corroborate that the above reported results depend on epistatic effects of CHRNA4 and DRD2 rather than on effects of the single genes, we analysed prediction accuracy for CHNRA4 and DRD2 alone (see S2 Table. With respect to CHRNA4, only a non-significant reduction in prediction error (MPRESS) was found when adding LV1 and LV2 to the model. Adding further latent variables overfitted the data (increase in MPRESS). With respect to DRD2, a significant improvement in the model was only found after adding the first LV, adding further LVs did not improve accuracy further. Note however, that overall classification accuracy is only slightly above chance level in this case.

In summary the PLS-DA approach yielded a network of attention-related brain regions whose responses to nicotine could be used to predict epistatic effects of CHRNA4/DRD2 genotype which cannot be predicted by CHRNA4 or DRD2 alone.

## Discussion

We investigated if gene by gene interactions of variations in CHRNA4 and DRD2 genes impact the effects of nicotine on reorienting visuospatial attention. On behavioural level, the strongest benefit of nicotine was observed in participants carrying both, the DRD2 T- and CHRNA4 C+ variant. Neurally, we were able to demonstrate that different DRD2/CHRNA4 groups can be decoded from the pattern of reorienting-related brain activity under nicotine. Neural substrates of interindividual variability were found bilaterally in the pulvinar, the striatum, the middle and superior frontal gyri, the insula, the left precuneus, and the right middle temporal gyrus. Our findings thus suggest that polymorphisms in the CHRNA4 and DRD2 genes are a relevant source of individual variability in pharmacological studies with nicotine.

To understand the contribution of genetic variation to the cognitive effects of nicotine we focussed on two well investigated SNPs in the CHRNA4 and DRD2 gene and grouped our subjects based on the suggested dominant roles of the C allele in the CHRNA4 gene and the T allele in the DRD2 gene. Markett et al. [13, 14, 19] revealed that behavioural and neuroanatomical phenotypes of the DRD2 gene are modulated by the presence of the C allele in the CHRNA4 gene. A dominant role of the CHRNA4 C allele was also observed in visuospatial attention tasks [7–10]. Hence, a pooling of C/C and C/T allele carriers of the CHRNA4 gene seems a plausible strategy when investigating the contribution of CHRNA4 genotype to visuospatial processes. The molecular mechanisms that lead to these different phenotypes in behaviour are not fully understood to date. It has been speculated that the CHRNA4 polymorphism (rs#1044396) determines the density of α4β2 nACh receptors in high-affinity states and thus might affect receptor responsiveness to ligands such as nicotine [9]. To our knowledge however, no changes in transcription rates, pre-mRNA splicing pattern, mRNA stability or receptor structure have been reported for CHRNA4 rs1044396. Behavioural evidence indicates however reduced attentional performance in C allele carriers which also exhibit an increased risk of nicotine addiction [9, 41]. In contrast to the sparse evidence on molecular and physiological consequences of the CHRNA4 rs#1044396 polymorphism, the DRD2 rs#6277 polymorphism is well investigated. In vitro studies suggest that the T allele is associated with reduced mRNA stability and DRD2 synthesis compared to the C-allele [11]. Since in vivo PET imaging provides evidence for higher striatal DRD2 availability, driven by alterations in receptor affinity and putatively dopamine level in T allele carriers, it has been suggested that the T allele is linked to a net impairment of DRD2 function [42–44].

On behavioural level, nicotine treatment accelerated overall reaction times and reduced the validity effect, which is in line with earlier studies that described enhanced attentional reorienting in non-smokers after nicotine [24, 38–40, 45]. As a new finding, we revealed that DRD2 and CHRNA4 genotype show epistasis effects associated with behavioural effects of nicotine on attentional reorienting. Only participants carrying both, the DRD2 T- and CHRNA4 C+ variant, showed a significant reduction of reaction times in invalid trials under nicotine. The finding that genotype dependent effects of nicotine treatment were only present in invalid trials is in line with other studies that investigated genetic polymorphisms in the DRD2 and CHRNA4 genes and that found effects in both genes predominantly in the more difficult or demanding task conditions [7, 10, 21]. Likewise, the reported epistasis effects of both genes in the studies by Markett and colleagues [14, 19] were primarily expressed during the more demanding task conditions.

Neurochemical interactions between dopaminergic and cholinergic signalling have primarily been investigated in the basal ganglia, where it has been suggested that nicotine-induced dopamine release via presynaptic receptors depends on a dynamic interplay between endogenous dopamine and acetylcholine levels [46]. The genetic polymorphisms investigated here may impact such dynamic interplay and modulate the effects of systemically administered nicotine. We have recently shown that DRD2 T-/CHRNA4 C+ subjects are also more responsive to the effects of nicotine on distractor interference, which corroborates the present findings [47]. A recent study further underlines the interactions between cholinergic and dopaminergic polymorphisms in showing epistatic effects of CHRNA5 and DRD2 on working memory and prefrontal cortex activity [48]. Taken together, it can be concluded that the attentional effects of nicotine reported here and in our prior study [47] are primarily seen in subjects with high dopaminergic activity (i.e. DRD2 T-) and a presumably higher rate of nicotinic receptors in high affinity state (i.e. CHRNA C+ according to Greenwood *et al*, 2012).

Neural substrates for genotype-dependent differences of nicotine were found bilaterally in fifteen brain regions including the pulvinar of the posterior thalamus, the striatum, the middle and superior frontal gyri, the insula, the left precuenus, and the right middle temporal gyrus. Or, put differently, variance of BOLD activity in those brain regions enabled to decode genotype with 87.23% accuracy. The identified brain regions overlapped either with neural circuits involved in visuospatial attention and/or are characterized by high densities of nicotinic ACh receptors and responsiveness to nicotinic stimulation in human pharmacological MRI studies. Nicotinic receptors in the primate brain are primarily of the α4β2 subtype, are located on presynaptic boutons and are highly abundant in the thalamus and striatum [49, 50].

Based primarily on early lesion studies, the pulvinar nucleus of the posterior thalamus was proposed as a central brain structure for orienting of attention [23]. This role was however not corroborated in later human neuroimaging studies and recent fMRI evidence indicates a role in distractor processing rather than (re)orienting of attention [51]. Distractor processing has been associated with variations in cholingergic neurotransmission. Prior behavioural evidence indicates that CHRNA4 C allele carriers are stronger influenced by distractors and that systemic administration of nicotine reduces distractor processing [9, 52]. As mentioned above we have recently extended these findings by providing evidence that the effects of nicotine on distractor interference depend on an interaction of the DRD2 and CHRNA4 polymorphism [47]. Even though the paradigm here did not involve distractors, we were able to show that the pulvinar is one of the brain regions where interindividual variability in response to nicotine can be used to decode CRHNA4/DRD2 genotype.

The striatum has often been related to reward processing and a prior pharmacogenetic fMRI study indicates that nicotine induced modulation of cortico-striatal activity depends on a functional genetic variation on the catechol-O-methyl-transferase (COMT) gene [53]. Several lines of evidence however suggest that the role of the striatum goes beyond reward processing since the caudate nucleus reacts to unexpected salient events even in the absence of reward [54]. Shulman et al. [55] suggested that the caudate nucleus forms part of a basal ganglia/frontal insula attention network that drives the dorsal attention network when reorienting is unexpected. We here show that neural activity in the striatum and insula contributes to genotype-dependent variability under nicotine. Complementary evidence by Markett et al. [19] suggests genotype-dependent differences in striatal gray matter density. Significant reductions in gray matter were found in participants carrying the DRD2 T- and CHRNA4 C- genes. Those participants with DRD2 T- / CHRNA4 C+ genotype who showed strongest behavioural effects of nicotine in the present study were characterized descriptively by higher striatal gray matter densities.

Prior fMRI studies that investigated the role of nicotine in cued target detection tasks have shown nicotinic modulations of reorienting-related neural activity in left and right middle frontal gyrus, right precuneus and left middle temporal gyrus [20, 24, 39, 40]. The right middle temporal gyrus was previously shown to react in a genotype-dependent way as a function of CHRNA4 genotype [56]. The present results extend these findings to a genotype dependent pharmacological modulation. CHRNA4 and DRD2 genes exerted epistasis effects on nicotine induced neural activity in these brain regions.

In summary, the multivariate PLS-DA approach was successful in revealing the neural circuitry underlying the genetic variability of nicotine’s attention enhancing effects. Since drugs acting on nicotinic cholinergic receptors have been suggested as potential treatment strategies for cognitive deficits in psychiatric disorders our findings also have potential translational implications. A limitation of the current study is however the small number of subjects and unequal group sizes which may be one reason why a conventional univariate approach was not sensitive enough to detect genotype-dependent effects of nicotine.

## Supporting Information

S1 Fig Frequency of voxels over VIP values (bin size = 0.02). Mean VIP values from the bootstrap procedure for all 105,476 voxels. 5% of the voxels (5,274) showed a VIP value which was equal or larger than 1.28, which was the VIP threshold used for the classification.
(PDF)

S2 Fig Comparison of identified brain regions relevant for genotype group differentiation (A, same figure as Fig 2 of the main manuscript) and conventional SPM ANOVA results at liberal threshold (B, threshold p<0.01, uncorrected; cluster extent threshold k≥40). (PDF)

S1 Table Behavioural Data: Mean reaction times (in ms) with standard error of the mean. (PDF)

S2 Table Classification performance for genotype groups (CHRNA4 & DRD2) and single genotypes (CHRNA4/DRD2 only). (PDF)
