# Supplementary material for: Nicotinergic Modulation of Attention-Related Neural Activity Differentiates Polymorphisms of DRD2 and CHRNA4 Receptor Genes
Source: PLoS One. 2015 Jun 16;10(6):e0126460. doi: 10.1371/journal.pone.0126460 (PMC4469651; doi:10.1371/journal.pone.0126460)
Supplement: S1 Fig — Mean VIP values from the bootstrap procedure for all 105,476 voxels. 5% of the voxels (5,274) showed a VIP value which was equal or larger than 1.28, which was the VIP threshold used for the classification. (PDF) [file pone.0126460.s001.pdf]

S1 Fig.

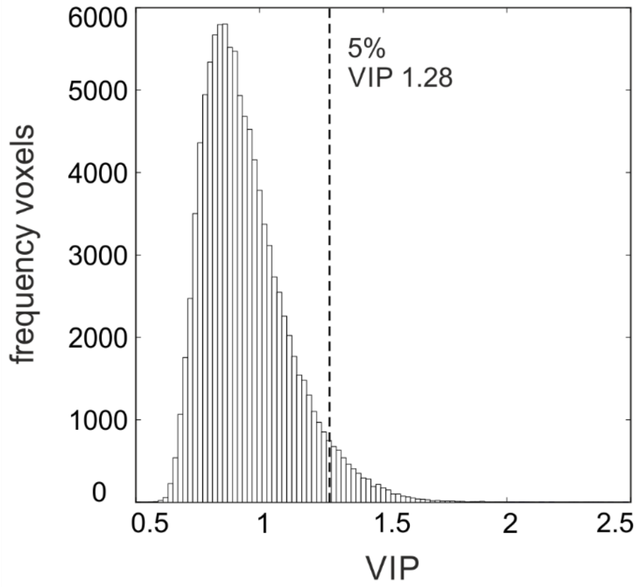

Supplemental Figure 1: Frequency of voxels over VIP values (bin size = 0.02). Mean VIP values from the bootstrap procedure for all 105,476 voxels. 5% of the voxels (5,274) showed a VIP value which was equal or larger than 1.28, which was the VIP threshold used for the classification.
