# Supplementary material for: Nicotinergic Modulation of Attention-Related Neural Activity Differentiates Polymorphisms of DRD2 and CHRNA4 Receptor Genes
Source: PLoS One. 2015 Jun 16;10(6):e0126460. doi: 10.1371/journal.pone.0126460 (PMC4469651; doi:10.1371/journal.pone.0126460)
Supplement: S2 Fig — (PDF) [file pone.0126460.s002.pdf]

S2 Fig.

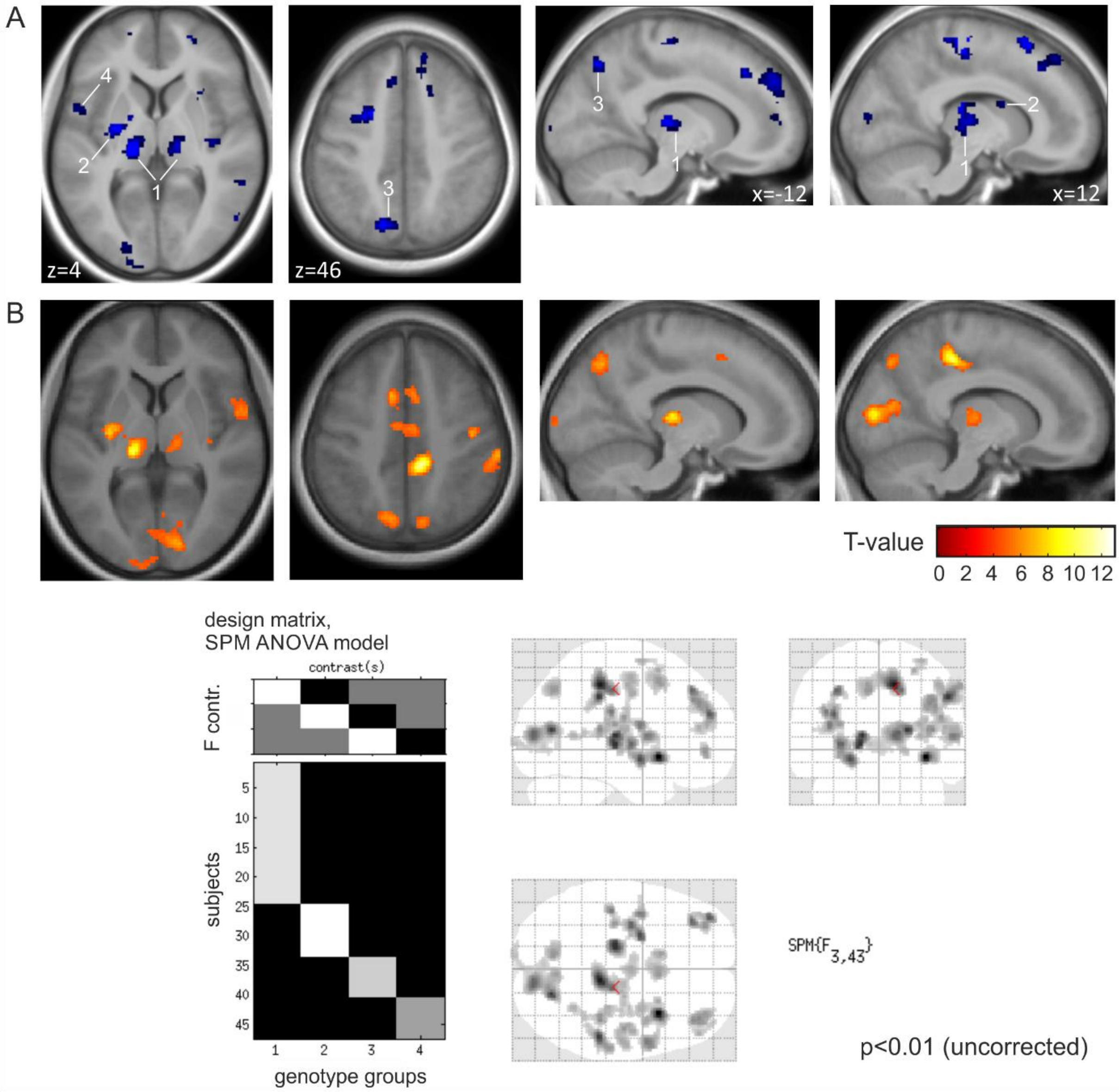

<u>Supplemental Figure 2:</u> Comparison of identified brain regions relevant for genotype group differentiation (A, same figure as Figure 2 of the main manuscript) and conventional SPM ANOVA results at liberal threshold (B, threshold $p<0.01$, uncorrected; cluster extent threshold $k \geq 40$).
