# Supplementary material for: Nicotinergic Modulation of Attention-Related Neural Activity Differentiates Polymorphisms of DRD2 and CHRNA4 Receptor Genes
Source: PLoS One. 2015 Jun 16;10(6):e0126460. doi: 10.1371/journal.pone.0126460 (PMC4469651; doi:10.1371/journal.pone.0126460)
Supplement: S1 Table — (PDF) [file pone.0126460.s003.pdf]

S1 Table

Behavioural Data: Mean reaction times (in ms) with standard error of the mean

	valid		invalid	
Genotype group	nicotine	placebo	nicotine	placebo
CHRNA4 C+ / DRD2 T+	362 (±7)	363 (±8)	431 (±7)	438 (±8)
CHRNA4 C- / DRD2 T+	340 (±12)	344 (±14)	435 (±14)	436 (±20)
CHRNA4 C+ / DRD2 T-	344 (±13)	356 (±15)	440 (±19)	480 (±30)
CHRNA4 C- / DRD2 T-	349 (±12)	355 (±13)	429 (±14)	437 (±12)
