# Supplementary material for: Nicotinergic Modulation of Attention-Related Neural Activity Differentiates Polymorphisms of DRD2 and CHRNA4 Receptor Genes
Source: PLoS One. 2015 Jun 16;10(6):e0126460. doi: 10.1371/journal.pone.0126460 (PMC4469651; doi:10.1371/journal.pone.0126460)
Supplement: S2 Table — (PDF) [file pone.0126460.s004.pdf]

S2 Table

Classification performance for genotype groups (CHRNA4 & DRD2) and single genotypes (CHRNA4 /DRD2 only)

	CHRNA4 & DRD2 [1]			CHRNA4 [2]			DRD2 [3]		
LV	MPRESS		Accuracy [%]	MPRESS		Accuracy [%]	MPRESS		Accuracy [%]
	p value	direction		p value	direction		p value	direction	
1	<0.01	(-)	65.96	0.07	(-)	68.09	0.04	(-)	57.45
2	0.01	(-)	65.96	0.26	(-)	65.96	0.08	(+)	46.81
3	0.02	(-)	72.34	0.14	(+)	63.83	0.64	(-)	46.81
4	0.02	(-)	87.23	0.23	(-)	72.34	0.58	(+)	46.80
5	0.33	(-)	89.36	0.33	(-)	74.47	0.25	(+)	55.32

[1]CHRNA4 & DRD2 (Data from the main article for comparison). Adding of LVs successively improve model prediction (decrease of MPRESS) and yield classification accuracies beyond chance level.

[2]CHRNA4. Adding LV1 and LV2 to the model reduces the prediction error (MPRESS), but not significantly. LV3 overfits the model (increase in MPRESS). Thus, further improvements in classification Accuracy (LV4 and LV5) are likely to be driven by noise.

[3]DRD2. LV1 significantly improves the model (MPRESS decrease), but leads to an overall classification accuracy that is only slightly above chance level. LV2 and succeeding LVs overfit and add noise to the model.
